# A Novel Distachionate from *Breynia distachia* Treats Inflammations by Modulating COX-2 and Inflammatory Cytokines in Rat Liver Tissue

**DOI:** 10.3390/molecules27082596

**Published:** 2022-04-18

**Authors:** Malik Saadullah, Muhammad Asif, Arshad Farid, Faiza Naseem, Sheikh Abdur Rashid, Shakira Ghazanfar, Muhammad Muzammal, Sohail Ahmad, Yousef A. Bin Jardan, Huda Alshaya, Muhammad Hamzah Saleem, Shafaqat Ali, Charles Oluwaseun Adetunji, Sania Arif

**Affiliations:** 1 Department of Pharmaceutical Chemistry, Government College University, Faisalabad 38000, Pakistan; maliksaadullah@gcuf.edu.pk (M.S.); arifsania456@gmail.com (S.A.); 2 Faculty of Pharmacy, Islamia University of Bahawalpur, Bahawalpur 63100, Pakistan; asif_pharmacist45@yahoo.com; 3 Gomal Center of Biochemistry and Biotechnology, Gomal University, Dera Ismail Khan 29050, Pakistan; mustafamuzammal1@yahoo.com (M.M.); sohailbiotech383@gmail.com (S.A.); 4 Department of Pharmaceutics, Faculty of Pharmacy, Gomal University, Dera Ismail Khan 29050, Pakistan; faizanaseem17@gmail.com; 5 National Institute for Genomics Advanced Biotechnology, National Agricultural Research Centre, Park Road, Islamabad 45500, Pakistan; shakira_akmal@yahoo.com; 6 Department of Pharmaceutics, College of Pharmacy, King Saud University, Riyadh 11451, Saudi Arabia; ybinjardan@ksu.edu.sa; 7 Department of Cell and Molecular Biology Program, University of Arkansas, Fayetteville, NC 72701, USA; hmalshay@uark.edu; 8 College of Plant Science and Technology, Huazhong Agricultural University, Wuhan 430070, China; saleemhamza312@webmail.hzau.edu.cn; 9 Department of Biological Sciences and Technology, China Medical University, Taichung 40402, Taiwan; shafaqataligill@yahoo.com; 10 Department of Environmental Sciences, Government College University, Faisalabad 38000, Pakistan; 11 Applied Microbiology, Biotechnology and Nanotechnology Laboratory, Department of Microbiology, Edo State University Uzairue, Iyamho 300271, Nigeria; adetunji.charles@edouniversity.edu.ng

**Keywords:** antioxidant, distachionate, lipoxygenase, cytotoxic, anti-inflammatory

## Abstract

*Breynia distachia* is a plant of genus Breynia belonging to family Phyllanthaceae. This study was conducted to isolate and examine the anti-inflammatory attributes of the roots of *Breynia distachia*. Methanol extract from roots were prepared by simple maceration. For phytochemical studies, isolation, purification, structure elucidation, metal analysis, total phenolic content, and solubility test were done by chromatographic and spectroscopic techniques. Anti-inflammatory activity was evaluated by cotton pallet edema model and carrageenan paw edema model, and antioxidant potential was evaluated by DPPH, FRAP, and ABTS antioxidants assays. Metal analysis of BD.Me revealed the presence of Na > Mg > K > Mn > Fe = Zn in respective order. Four phytochemicals such as gallic acid, quercetin, sinapic acid, and *p*-coumaric acid are found in *Breynia distachia*. Quercetin is present in relatively larger quantity, and shows antioxidant activity by reducing the ferric iron to ferrous iron. Novel distachionate shows high antioxidant activity in ABTS assay by reducing reactive oxygen species. Quantitative or qualitative analysis performed by HPLC indicates the ascending peaks or presence of secondary products (metabolites) respectively. Histopathology analysis of liver, spleen, heart, and kidney was done, revealing mild inflammations in spleen and liver, and no cytotoxicity in heart and kidney. Oral administration of BD.Me and ditachionate significantly inhibits the carrageenan and cotton pellet-induced paw edema in 1st and 2nd h with (ns = *p* > 0.05) than control. After 3rd, 4th, 5th, and 6th h, BD.Me and ditachionate showed inhibition of paw edema in a highly significant (*** = *p* < 0.001) manner as compared to control. In cotton-pellet edema model, distachionate shows a %inhibition of 57.3% at a dose level of 5 mg/kg. Docking values obtained from distachionate-COX-2 complex suggest a potent inhibitor evaluated for this protein. The distachionate shows effective anti-inflammatory activity. Methanol extracts of roots showed significant lipoxygenase inhibitory activity by IC_50_ values of 155.7 ± 0.55 and 132.9 ± 0.33 μg/mL. Data from various in vitro and in vivo models suggest that novel distachionate isolated from *Breynia distachia* shows strong antioxidant and anti-inflammatory activities; it should be further studied for the exploration of its medicinal potential.

## 1. Introduction

Mechanical stimulation, microbial invasion, and irritants can all produce inflammation, which is a complex biological reaction to tissue injury. Cyclooxygenase-2 (COX-2) is an enzyme of prostaglandins, produced in response to inflammation. COX-2 production supports the process of inflammation and mediates pain. The nonsteroidal anti-inflammatory drugs (NSAIDs) selectively inhibits the COX-2 enzyme, hence inhibiting inflammation [1]. The inflammatory cytokines of the spinal cord, dorsal root ganglion, and injured nerves are associated with pain behavior. Cytokines are the proteins that are secreted by cells and have specific effect on the interaction of cells. They include lymphokine, monokines, chemokines, and interleukin. The activated macrophages produced inflammatory cytokines. The pathological pains are induced by IL-1β, IL-6, and TNF-α cytokines [2]. 

All medicinal plants contained secondary metabolites. These metabolites are helpful for the treatment of different diseases. They show antioxidant, angiogenic, antimicrobial, anti-inflammatory, and cell signaling effects that are vital contributors in wound healing process. They are classified according to their composition, chemical structure, solubility in various solvents, and according to their synthetic pathway. They are broadly classified into phenolics, terpenes, alkaloids, steroids, and flavonoids. All secondary metabolites show large biological activity, 25% of molecules used in pharmaceutical industry are of plant origin [3].

*Breynia distachia* is a plant of genus *Breynia* belonging to family Phyllanthaceae and consist of about 35 species which are distributed from India to Australia. The different constituents isolated from the genus are friedelan-3b-ol, friedelin, aviculin (+)-isolariciresinol-9-rhamno-pyranoside, isoarborinol, arborinone, 5-hydroxy-7,8,4-trimethoxy, 2,4-dihydroxy-6-methoxy-3-methyl acetophenone, and flavone respectively [4]. *Breynia distachia* is a shrub having purple, green, and white color leaves and reported to have antioxidant activity. Breynin I (a new sulfur-containing spiroketal glycoside) and Breyniaionoside E (a new terpenic glycoside) were isolated from the aerial parts of *Breynia* [5]. Due to the existence of secondary metabolites (glycosides, terpenoids, alkaloids, lignans, steroids, nucleosides, tannins, and phenolic), the genus *Breynia* has medicinal potential. 

Based on these facts this study aims to evaluate the anti-inflammatory and antioxidant potential of novel distachionate isolated from *Breynia distachia* in wound healing by modulating COX-2 and inflammatory cytokine in rat’s liver. A series of in vivo and in vitro assays are performed. Moreover, possible mechanism of action with COX-2 will also be explored by the help of docking data. Results suggest that *Breynia distachia* extract and isolated compound i.e., distachionate has a strong anti-inflammatory impact and can be used to treat inflammation as well as other diseases.

## 2. Results

Percent yield value of BD.Me was calculated to be 5%.

### 2.1. Phytochemical Analysis

#### 2.1.1. Solubility Analysis

BD.Me was found to be soluble in aqueous, methanolic, and ethanolic solvents by vortex mixing.

#### 2.1.2. Metal Analysis

BD.Me (50 mg) showed the presence of iron, manganese, potassium, zinc, sodium, and magnesium, concentration (mg) of each metal present in 50 mg of BD.ME is mentioned in Table 1. 

### 2.2. Phytochemical Characterization

#### Quantitative Analysis by HPLC

**Figure 1:**
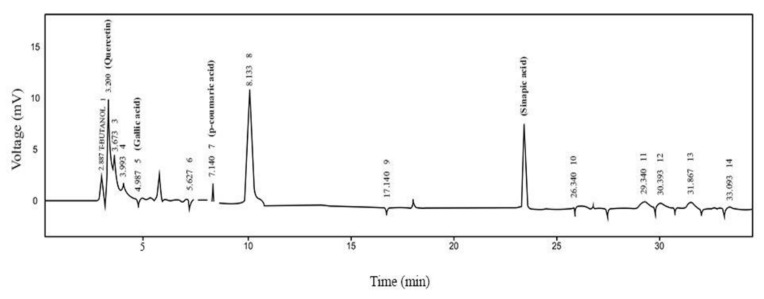
HPLC chromatogram of methanolic extract of *Breynia distachia.*

Major four phytochemicals, gallic acid, quercetin, sinapic acid, and *p*-coumaric acid are found in BD.Me that belongs to polyphenols, phenolic acid, and phenylpropanoid class respectively. Quantitative analysis indicated that major ascending peak corresponds to quercetin (13.8%) followed by gallic acid (2.4%), sinapic acid (0.8%), and *p*-coumaric acid (2.3%), Figure 1.

**Figure 2:**
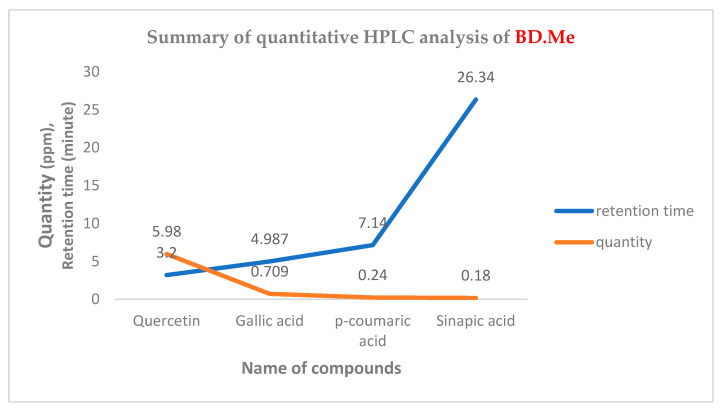
Summary of quantitative HPLC analysis of BD.Me.

Data in Figure 2 show retention time and quantity (ppm) of four phytochemicals identified through HPLC analysis. BD.Me has the highest amount of quercetin flavonoid (5.98) followed by, sinapic acid, *p*-coumaric acid, and gallic acid respectively.

### 2.3. Qualitative Analysis

Secondary metabolites such as alkaloids, phenols, glycosides, saponins, and tannins were found in BD.ME as a result of phytochemical investigation, whereas primary metabolites carbohydrates, proteins, and flavonoids were found to be absent. Results are mentioned in Table 2.

#### Total Phenolic Content

Total phenolic content in BD.Me was calculated to be 0.44 ± 0.12 μmol/mL.

### 2.4. Purification of Novel Compound

**Figure 3:**
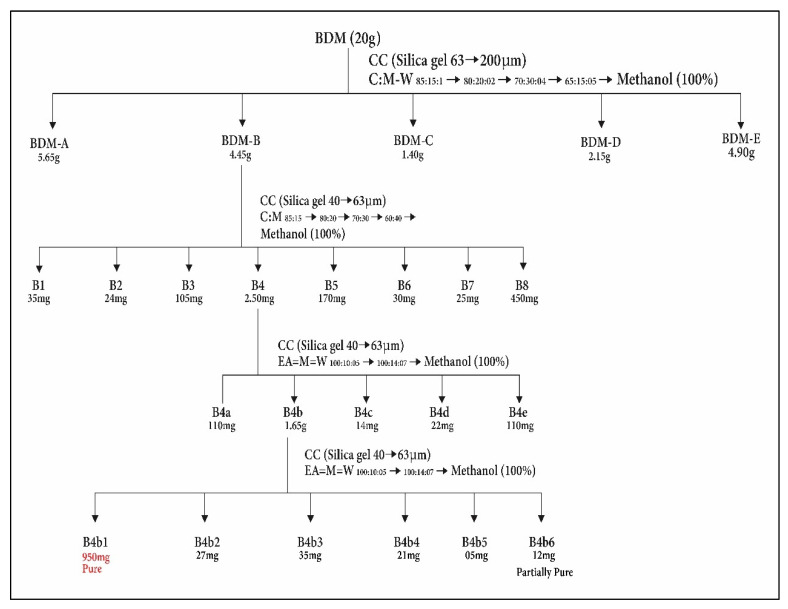
Isolation scheme of distachionate from *Breynia distachia*.

The fractionation of methanol extract of *Breynia distachia* (BD.ME) 20 g was carried out by using open column chromatography, stationary phase (silica gel 60 (63–200 μm)) and mobile phase (chloroform, methanol, water), processed by step-wise elution with increased polarity. The five fractions were made as BDM-A to E. On the basis of TLC analysis, the fraction BDM-B of 4.45 gm was further processed by column chromatography using the ratio (85:15:1 chloroform:methanol:water) as mobile phase and silica gel 60 (40–63 μm) as stationary phase. Total of eight fractions were obtained such as B1 to 8. TLC analysis was performed once more for all the eight fractions. Total of 2.50 g of the fraction B4 was examined further by column chromatography with ethyl acetate:methanol:water 100:10:5 as the mobile phase ratio. By this process, five fractions were obtained (B4-a to B4-e). The B4-b fraction of 1.65 gm was fractioned (again) by column chromatography with ethyl acetate:methanol:water at ratio of 100:10:5 as mobile phase and silica gel 60 of 40–63 μm as stationary phase. Six fractions were obtained (B4b-1 to B4b-6). The fraction B4b-1 (950 mg) was obtained as pure compound. The purification scheme is given in Figure 3. 

### 2.5. Structure Elucidation

**Figure 4:**
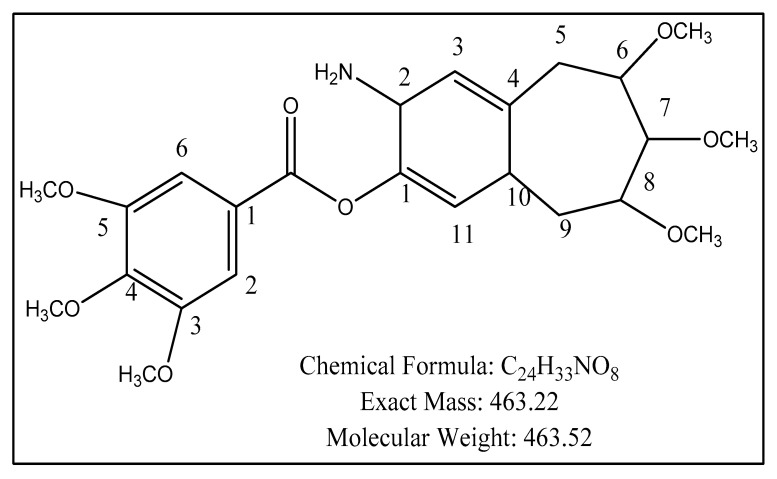
Structure of distachionate.

Distachinoate (Figure 4) was obtained as yellowish powder from methanol extract of *Breynia distachia*. The molecular weight was 463.22 with chemical formula C_24_H_33_NO_8_, which was illucidated by the use of HR-EI-MS showing ion peak at *m*/*z*, 463.22. The aromaticity in a compound was illucidated by the IR spectra at 2834.5 cm^−1^, 1698.5 cm^−1^ for (C = 0), and 1520.2 cm^−1^ wavelength absorption. The stretching at 2754 cm^−1^ was due to Sp2 hybridization. The conjugation in structure was observed by ultraviolet spectrum which absorbs radiation at 244 and 259 nm.

The ^1^H-NMR (Table 3) of distachinoate revealed aromatic protons at 6.85 δ7.54 s (1H, dd, *J* = 2.1 Hz), 6.97 s (d, *J* = 2.0 Hz), 7.30 (dd, *J* = 0.4 Hz), 6.90 s (1H, *J* = 1.02 Hz), 6.20 (1H, d, *J* = 1.45 Hz). The lactone proton signals were shown at δ 3.95 (1H, d, *J* = 2.1, 3.4 Hz). The signals at δ 4.40 (s for 1H) in downfield are alkenes. The protons, which appear at δ 1.92 and δ 1.78, were diastereopic in nature. 

The mass spectrum of distachionate shows retro Diels Alder fragmentation pattern by EIMS peak having *m*/*z* that confirms three methoxyls moieties in ring B and three methoxyl moieties in ring A. The ^13^C-NMR (broad band) (Table 3) spectra of distachionate shows twenty-four signals of carbon for methines (8), methylenes (10), and quaternary (8) carbons. The aromatic carbons give the signals at δ 120.4, 121.6, 130.2, 131.1, 129.3, and 128.2. The singlet at δ 139.2 shows the presences of carbonyl carbon in a molecule. The signals at 30.2 and 34.1 revealed the presence of aliphatic carbons. The structure was verified by observing the HMBC correlations from H-15, H-18, H-21, and H-22 (δH, 1.85 m) to C-17, and H-15 to C-16. 7, 8, and 9 were deduced from the chemical shifts, similar coupling constants, and NOESY correlations.

All indicated data of spectral and physical values show that the structural elucidation of compound as 2-amino-6,7,8-trimethoxy-4a,5,6,7,8,9-hexahydro-2*H*-benzoannulen-3-yl 3,4,5-trimethoxybenzoate (novel natural product). Distachionate name was given on the basis of species name.

### 2.6. Antioxidant Assays

#### In Vitro

Data in Table 4 indicated moderate radical scavenging potential of distachionate and BD.Me by DPPH and ABTS assay, while FRAP assay showed good antioxidant power. IC_50_ values of standard and BD.Me are compared in Table 4.

### 2.7. In Vivo

#### Antioxidant Enzymes

Data in Table 5 revealed that total TSP (tacrolimus, sirolimus, prednisone drugs) level was elevated whereas significant decrease of SOD (suboxide dismutase enzyme) and CAT (catalase enzyme) in control group as compared to standard, BD.Me and distachionate-treated group.

### 2.8. Histopathological Results

**Figure 5:**
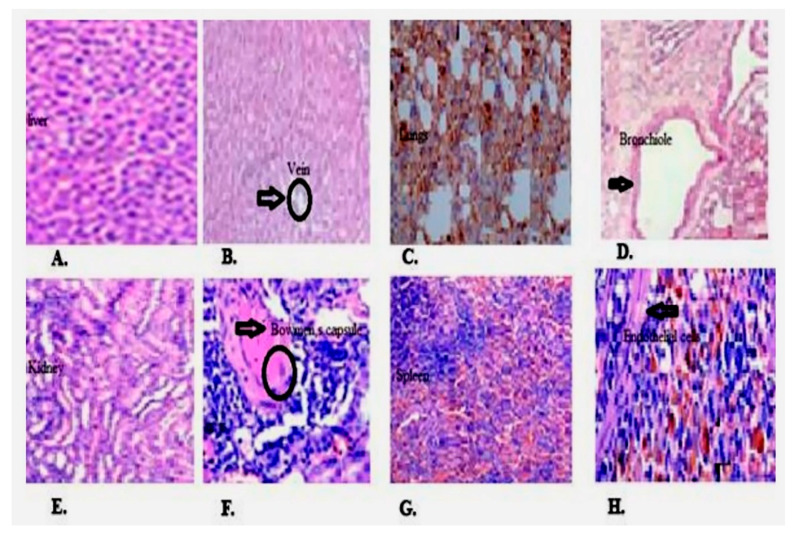
Histopathological microscopic image of liver, lung, kidney, and spleen by hematoxylin and eosin (H & E) stain (magnification-10×). (**A**,**B**) Liver shows mild focal inflammation and ceroid macrophages (black arrow head), (**C**,**D**) mild focal hemorrhage in BD.Me-treated group and diffuse hemorrhage in both groups (black arrow head), (**E**,**F**) shows mild interstitial inflammation (black arrow head), (**G**,**H**) shows excessive hemosiderin deposition (black arrow head) in endothelial cells and expansion of red pulp in spleen in BD.Me-treated group.

Histopathological analysis of liver, heart, lung, kidney, and spleen is shown in Figure 5, mild focal inflammation is observed on liver. Expansion of red pulp and excessive hemosiderin deposition are seen in spleen in treatment group as compared to control. Lungs have shown diffuse hemorrhage while no renal and cardiotoxicity are examined.

### 2.9. Wound Healing Potential of BD.Me

Data in Table 6 indicates the accelerated effect of BD.Me on wound healing process. In wound closure treatment on 3rd day the mean ± SEM values of control was 1.72 ± 0.61 while 10% BD.Me and 20% BD.Me cream treatment increased the effect by 3.85 ± 0.85 and 6.46 ± 0.98 respectively than control. 20% BD.Me cream showed increase in % wound contraction non-significantly (ns = *p* > 0.05) as compared to 10% BD.Me cream. Standard showed highly significant (*** = *p* < 0.001) wound contraction than control whereas with comparison of 20% BD.Me cream standard produced non-significant (ns = *p* > 0.05) effect. On 7th, 10th, 14th and 21st day wound contraction was increased in standard, 20% BD.Me cream, 10% BD.Me cream, and control respectively.

Table 6 and Table 7 show dose dependent effect of distachionate and BD.Me on skin in 0, 3rd, 7th, and 14th day in excision model and 0, 3rd, 7th, 14th, and 21st day in burn wound healing model, four experimental rat groups respectively.

### 2.10. Carrageenan Paw Edema Model

The edema induced by carageenan is significantly (*p* < 0.05) inhibited by BD.Me and novel isolate distachinoate. The 5 mg/kg dose of distachionate and BD.Me shows highly significant (*p* < 0.05 and *p* < 0.01) inhibition with 58.25% of granuloma inhibition in 5th h of administration. Percentage inhibition increases from 1st h to 5th h of administration.

### 2.11. Cotton-Pellet Edema Model

The BD.Me extract and distachinoate have significant (*p* < 0.05) inhibition in cotton pellets induced edema. The 5 mg/kg dose of distachionate and BD.Me shows highly significant (*p* < 0.05 and *p* < 0.01) inhibition with 52.42% of granuloma inhibition in 5th h of administration.

### 2.12. Statistical Analysis of Anti-Inflammatory Study

**Figure 6:**
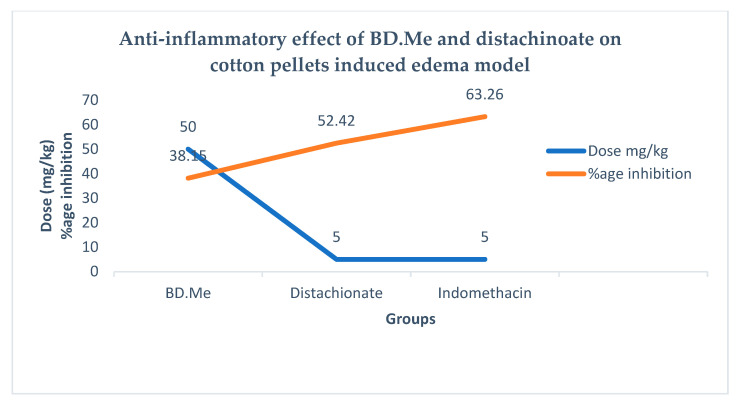
Anti-inflammatory effect of *Breynia distachia* methanol extract and distachinoate on cotton pellets induced edema model (mean ± SEM): *p* < 0.05, *p* < 0.01 compared with negative control group receiving saline.

Data in Table 8 and Figure 6 depict significantly inhibited effect of orally administered distachionate and BD.Me on carrageenan and cotton pellet-induced paw edema. After 1st and 2nd h of carrageenan injection, 100 mg/kg and 200 mg/kg BD.Me treated group showed non-significant (*p* > 0.05) inhibition of paw edema than control. After 3rd, 4th, 5th, and 6th h, plant extract at dose 100 mg/kg, 200 mg/kg, and indomethacin at 10 mg/kg showed inhibition of paw edema in a highly significant (*p* < 0.001) manner as compared to control. In cotton pellet model, elevation in edema level was observed continuously in control group than all other groups throughout the 14 days period. Mean ± SEM value of BD.Me at dose level 100 mg/kg and 200 mg/kg and standard group showed dose-dependent significant inhibition of edema. For graphical representation see Figure 6.

The histopathological findings of skin biopsy in punch wound healing study and burn wound healing study are mentioned in Table 9 and Table 10, respectively. These findings indicate the wound healing and intensity of inflammation, which indicates wound progression in different groups.

### 2.13. Docking

**Figure 7:**
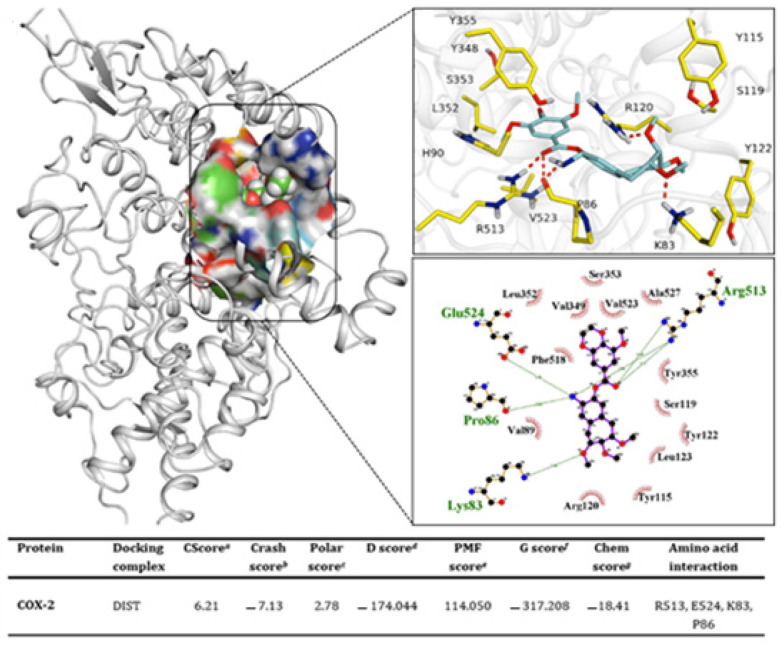
The docking conformation exhibit the binding interactions of DIST-COX-2 complex favorable for its attachment to the protein. *^a^* CScore is a consensus scoring system that ranks the affinities of ligands using a variety of scoring mechanisms. *^b^* Crash-score indicating inadvertent penetration of the binding site, *^c^* Polar region of the ligand, *^d^* D-score demonstrating complex (ligand-protein), internal (ligand-ligand) energies and hydrogen bonding, the Helmholtz free energy of interactions for protein-ligand atom pairs are indicated by the *^e^* PMF-score (Potential of Mean Force, PMF), *^f^* G-score shows van der Waals interactions and charge between ligand and protein, *^g^* Chem-score indicates the lipophilic contact, hydrogen bonding and rotational entropy, along with intercept term.

Molecular interaction pattern was obtained as a result of protein–ligand binding. The best pose from docked complexes was used to demonstrate the effective inhibitory pattern for the ligand. The ligand exhibited its greatest affinity toward the target and exhibited the best docking scores (CScore: 6.21) (Figure 7). Arg513 emerged as the potent residue responsible for their binding to the ligand. Arginine has a profound role due to its strong binding interaction exhibited through three intermolecular hydrogen bonds. It could be suggested that this residue has the primary role in the binding for ligand. Glu524 and Pro86 and Lys83 are the other residues involved in forming the binding interaction. The Glu524 and Pro86 residual interaction was found to be sharing common ligand atom as Arg513. Docking values obtained from distachionate-COX-2 complex suggested a potent inhibitor evaluated for this protein.

## 3. Discussion 

By using in vitro and in vivo models, the current study shows anti-inflammatory and antioxidant effects of BD.Me (methanolic extract of *B. distachia*) for the first time. Before in vivo experiments, metals are necessary for the maintenance of physiological and biological processes in the human body, so the extract was tested for the presence of many metals. Increases in their intake (above at specific permissible limits (PL)) can cause toxicity [6]. Metal analysis revealed the presence of Na > Mg > K > Mn > Fe = Zn in respective order. In observational studies, excessive sodium intake has been linked to inflammation in people with high blood pressure and atherosclerosis, while decreased salt intake has been linked to lower mortality, heart disease, and stroke. A high-sodium diet has been linked to elevated inflammatory signs in one small human investigation [7]. Mg has a strong anti-inflammatory impact. Magnesium deficiency inhibits the release of inflammatory mediators [8]. As a result, sodium, magnesium, potassium, zinc, iron, and manganese found in BD.Me are thought to contribute to its anti-inflammatory properties. Secondary metabolites such as alkaloids, glycosides, phenols, saponins, and tannins were found in BD.Me after phytochemical examination, whereas primary metabolites carbohydrates, proteins, and flavonoids were found to be absent. The COX-2, TNF, and IL are all inhibited by alkaloids, resulting in anti-inflammatory effects. Phenols have antioxidant and anti-inflammatory effects [9]. They block NF-KB, which inhibits iNOS, COX-2, LOX, and the production of inflammatory mediators (PGs, IL, and TNF) [10]. Glycosides prevent the synthesis and release of TNF, and IL-1, so they have anti-inflammatory properties [11]. Saponins are also thought to have anti-inflammatory properties [9]. They inhibit the COX-2 enzyme, resulting in lower levels of PGE2, NO, and TNF-α, resulting in anti-inflammatory effects [12]. In wound healing the tannins are involved and have analgesic, astringent, antibacterial, antifungal, antioxidant, and anti-inflammatory properties [9]. Antioxidant potential of *B. distachia* are evident by our findings of HPLC analysis (reveals that five different compounds are present). In the present study, HPLC analysis of BD.Me revealed the presence of quercetin with maximum peak area. The wound healing is enhanced by quercetin because it regulates cytokines, growth factors, and cells involved in the inflammatory and proliferative phases of the healing process [13]. Other phenolic compounds include *p*-coumaric acid, gallic acid, sinapic acid which show antioxidant and anti-inflammatory activities by inhibiting COX-2 enzyme and by reducing NO, PGE2, and IL-6 levels. The damage caused by ROS and free radiclas is prevented by antioxidants [14]. Three antioxidant assays were performed for the assessment of scavenging activity of BD.Me and distachionate against different free radicals. The DPPH and ABTS assays are used for the measurement of antioxidant activity against cation free radicals and stable organic nitrogen, respectively, whereas antioxidant capacity is measured in the FRAP assay by ferric ion reduction. In DPPH and ABTS assays, low IC50 shows higher antioxidant potential, whereas in the FRAP assay, high reducing power shows high antioxidant potential. In the present study, the BD.Me and distachionate showed high free radical scavenging activity in these in DPPH, ABTS and FRAP assays. BD.Me shows higher scavenging activity than distachionate. Furthermore, most essential antioxidant enzyme of mitochondria is SOD, and it protects against superoxide anion. Neutrophil-mediated inflammation is inhibited by the SOD enzyme [15]. This study reveals that BD.Me and distachionate treatment increased the levels SOD and CAT, suggesting that the reduction in inflammation by reducing reactive oxygen species (ROS). Catalase is another oxygen-scavenging antioxidant and defense enzyme [16]. In carrageenan-induced paw edema the release of inflammatory mediators is biphasic. In the first phase histamine, serotonin, and bradykinin were released, and in second phase prostaglandins (PGs), cytokines, TNF-, IL-1, and IL-6 were released [17]. In the present study, during the early hours of BD.Me and distachionate dosing show significant suppression in paw edema, but more effective in later hours. In the treatment with BD.Me and distachionate, carrageenan-induced paw edema was significantly reduced. After 2 h of injection, distachionate and BD.Me show inhibition (significant) of paw edema induced by carageenan. The IL-6 (inflammatory marker) works by acute phase proteins generation, have impact on B and T cells, boosts the VEGF expression, an angiogenesis promoter [18]. The pro-inflammatory cytokine (TNF) stimulates oxidative damage at the inflammatory site and leukocyte recruitment to the epithelium [19]. The level of TNF- and IL-6 in rat’s serum (treated with BD.Me and distachionate) were significantly reduced. At the site of inflammation the number of inflammatory cells were significantly decreased, which has been postulated as a possible cause. WBC migration is involved in cotton pellet-induced granuloma, which is a proliferative stage of inflammation. It is a standard method for determining anti-inflammatory potential during chronic inflammation. Monocyte filtration, fibroblast proliferation, angiogenesis, and exudation are all involved [20]. The inflammatory mediators in cotton pellet-induced inflammation are inhibited by the treatment of BD.Me and distachionate, implying that it inhibits the release and action of inflammatory mediators. Overall, distachionate and BD.Me show anti-inflammatory and antioxidant activities by inhibiting COX-2 and cytokines.

## 4. Materials and Methods

### 4.1. Plant Collection

*Breynia distachia* (whole plant) was collected from Bahauddin Zakariya University Multan (botanical garden) and the roots were separated. A voucher specimen Stewart EC-233 was deposited in herbarium of Bahauddin Zakariya University, Multan, Pakistan.

### 4.2. Plant Extraction

After collecting the plant, dust, debris, and insects are removed from the 3 kg fresh plant, which is then thoroughly cleaned with tap water, air dried for 17 days, and ground into powder in an electrical grinder. For extraction, simple maceration technique was performed on plant powder. Methanol (95% absolute, sigma Aldrich, Burlington, MA, USA) (1 L/kg) was added in weighed amount (2.8 kg) of plant powder, on daily basis the container having mixture was kept in sonicator (grant ultrasonic bath Findel international E8RO5651, National High-tech Enterprise, Longhua, Shenzhen, China) for 30 min. After 7 days, filtration was done with Whatman grade 1 filter paper, repeated this process for three times and the filtrate was evaporated on rotary evaporator at 40 °C leaving behind greenish residue, stored in Petri dish for two days at room temperature (15–30 °C). Total of 25 g of extract was obtained and labeled as BD.Me. For further experiments, the extract was collected in an airtight container and stored at 4 °C at a safe place [21].

### 4.3. Phytochemical Analysis

Reported method was used for analysis of primary and secondary metabolites by the defined procedure [22]. See Supplementary Materials for details.

### 4.4. Solubility Test

For solubility test the 10 mg of BD.Me extract was dissolved in 1 mL of distilled water, gum acacia, normal saline, ethanol, and methanol (50%, 100%) with the help of a vortex mixer and heat.

### 4.5. Trace Metal Analysis

GUVEN and Akinci 2011 method was used for analysis of trace metal [23]. See Supplementary Materials for details.

### 4.6. Total Phenolic Content Assay

For total phenolic content analysis Folin–Ciocalteu method was used [24], detail is given in Supplementary Materials.

### 4.7. Phytochemical Characterization

#### 4.7.1. High Performance Liquid Chromatography (HPLC)

By using HPLC the total phenolic and flavonoids content of the test drug was determined. The Shim pack HPLC CLC-ODS (C-18) column with 25 cm × 4.6 mm length and width, 5 μm diameter was used. In this method, two types of mobile phases were used. Mobile phase A was acetonitrile, run as 15% for 0–15 min, 45% for 15–30 min, 100% for 30–45 min, whereas mobile phase B contained distilled water:acetic acid (94:6) and the pH was adjusted to 2.27. A UV visible (UV) detector with a wavelength of 280 nm was employed to detect the sample at room temperature. The chromatogram was generated by plotting voltage on x-axis and time on y-axis. By the comparison of reference standards and sample peaks, the compounds were identified. Instrument of HPLC was made of Shimadzu, Kyoto, Japan (detector SPD-10 AV; pump. LC-10 AT) [25].

#### 4.7.2. Thin Layer Chromatography

Thin layer chromatography process was done as following procedure [26]. See details in Table S1 of Supplementary Materials.

#### 4.7.3. Column Chromatography

In column chromatography, silica gel (particle size 63–200 μm) was used as the stationary phase in the column of 25 cm in length and 3.4 mm–4.6 mm diameter. In mobile phase the chloroform, methanol, and water with 85:15:1, 80:20:2, 65:35:5 ratios and 100% methanol in sufficient quantity were run through the column. Step-wise elution is done with these mobile phases. The compounds of plant extract run down and form band based on the polarity and molecular nature of these compounds. Then the eluted volumes are collected sequentially as fractions. 

### 4.8. Purification

After isolation with column chromatography, the purification of bioactive compound was done with the help of HPLC which accelerate the process of purification. In HPLC analysis, a 0.45 m Millex-HV PVDF membrane (Millipore, New Bedford, MA, USA) was used to filter the sample. The chromatographer (Shimadzu chromatographer) with DAD detector (diode assay detector) and ternary pump (Shimadzu LC-20AT, Kyoto, Japan) (Shimadzu SPD-M20A, Kyoto, Japan) was used for HPLC analysis. It is carried out on a Phenomenex ODS 100 A 250 mm 4.60 mm, 5 m analytical column, which is preceded by a C18 guard column (2.0 cm 4.0 mm; 5 m). For data processing, the LC solution software of 1.25 version was used. For mobile phase the acetonitrile and water with gradient chromatographic conditions were used. Initially, 2:8 *v*/*v* of acetonitrile and water were used, then after 30 min the level was increased to 8:2 *v*/*v* with 1 mL/min flow rate. The temperature and volume of the column were maintained at 25 °C and 20 μL respectively. At the range of 450 to 200 nm the UV spectra were measured. The methanol was used for the preparation of all solutions (standard and extracts), at six concentration the standard solutions were used (12.5, 25.0, 100.0, 150.0, 250.0, and 500.0 gm/L), while the solution compound concentration was 2000 gm/L.

### 4.9. Structure Elucidation

The chemical structure of compound was elucidated with the help of two-dimensional correlation techniques (HMBC, HSQC), UV-visible and infrared spectroscopy (IR), proton nuclear magnetic resonance (1H-NMR), 13C-NMR (BB, DEPT-135, 90), and mass spectrometry [27].

### 4.10. Pharmacological Assays

#### 4.10.1. Antioxidant Assays

For antiradical assays, recognized techniques of DPPH, ABTS, and FRAP assay were used [28]. See Supplementary Materials for details.

#### 4.10.2. Antioxidant Enzymes

In vivo antioxidant enzymes study was performed for quantification [29]. For details, see Supplementary Materials.

### 4.11. Pharmacological Activities

#### 4.11.1. Acute Toxicity Study

##### Animals

Animal studies protocols and their handling were approved by ethical committee of Government College University Faisalabad, with reference number GCUF/ERC/201 and study #20136.

##### Experimental Design

According to OECD 425, Guidelines of Chemical Testing and acute toxicity was performed by following the procedure [30]. See details in Supplementary Materials.

### 4.12. Wound Healing Study

#### 4.12.1. Study Design

Twenty-four healthy albino rats having weights 200–250 g were randomly selected irrespective of gender for every model. See Supplementary Materials for details.

#### 4.12.2. Excision Wound Healing Model

Excision wound healing model of Jahandideh, Hajimehdipoor et al. (2017) [31] was used. See Supplementary Materials for details.

#### 4.12.3. Burn Wound Healing Model

Well-established protocol with slight modifications was used as described in the following procedure [32]. Detail is given in Supplementary Materials.

### 4.13. Anti-Inflammatory Study

#### 4.13.1. Carrageenan Paw Edema Model

Reported method of Shabbir, Batool et al. (2018) [33] was used, see Supplementary Materials for details. 

#### 4.13.2. Cotton Pellet Edema Model

A well-established method of Owoyele, Adebukola et al. (2008). [34] with slight modification. See Supplementary Materials for details.

## 5. Conclusions

This study was designed to evaluate the antioxidant and anti-inflammatory potential of *B. distachia* in wound healing and its uses in a series of in vivo and in vitro assays. Moreover, possible mechanism of action will also be explored by the help of docking data. The distachionate compound showed active inhibitory activities for enzymes, and the reduction of ferric to ferrous shows its antioxidant property.

## Figures and Tables

**Table 1:** Metal analysis of plant material of *Breynia distachia.*

Sr. No.	Name of Element	Dry Powder/*Breynia distachia*’s Plant Material (50 mg)
1	Iron (Fe)	0.004 mg
2	Manganese (Mn)	0.005 mg
3	Potassium (K)	0.830 mg
4	Zinc (Zn)	0.004 mg
5	Sodium (Na)	4.056 mg
6	Magnesium (Mg)	2.357 mg

**Table 2:** Qualitative analysis of phytochemicals.

Phytochemicals	Presence or Absence
Alkaloids	+ve
Phenols	+ve
Glycosides	+ve
Saponins	+ve
Tannins	+ve
Carbohydrates	−ve
Proteins	−ve

**Table 3:** ^13^C-NMR and ^1^H-NMR of distachionate.

Carbon No.	Multiplicity DEPT	^13^C-NMR (δ)	^1^H-NMR	*J*-Value
C-1	C	143.8	-	-
C-2	CH	53.4	3.99 dd	1.6 Hz
C-3	C	114.3	-	-
C-4	C	137.2	-	-
C-5	CH_2_	34.3	2.21, 1.96 d	3.2 Hz
C-6	CH	82.2	3.12	3.0 Hz
C-7	CH	94.43	3.20 d	2.4 Hz
C-8	CH	84.25	2.99 dd	2.6 Hz
C-9	CH_2_	30.4	1.50, 1.23 d	3.50 Hz
C-10	CH	31.55	2.50 dd	2.1 Hz
C-11	CH	109.65	4.74 d	3.25 Hz
C-12	C	121.2	-	-
C-13	CH	104.45	7.1	2.20 Hz
C-14	C	149.26	6.85 d	-
C-15	C	139.15	6.90 s	-
C-16	C	148.5	7.54 s	-
C-17	CH	104.33	6.97 s	1.60 Hz
C-18	C	159.25	-	-
C-19	OCH_3_	54.33	3.80 s	-
C-20	OCH_3_	57.2	3.82 s	-
C-21	OCH_3_	54.62	3.90 s	-
C-22	OCH_3_	55.35	3.18 d	5.2 Hz
C-23	OCH_3_	55.62	3.21 d	4.3 Hz
C-24	OCH_3_	56.1	3.22 d	4.0 Hz

**Table 4:** Free radical scavenging activity of BD.Me by different antioxidant assays.

Antioxidant Power	Sample		
	Ascorbic Acid (Standard)	BD.Me	Distachionate
ABTS IC50 (μg/mL)	3.33 ± 1.07	60.85 ± 5.1	30.75 ± 3.2
DPPH IC50 (μg/mL)	2.93 ± 0.32	79.50 ± 2.64	23.10 ± 2.40
FRAP (nmol Fe + 2 per mg extract)	-	390.13 ± 14.25	28.25 ± 3.50

Values shown are mean ± SD of BD.Me antioxidant assays.

**Table 5:** Effect of BD.Me on antioxidant enzymes.

Antioxidant Enzymes	Effect of Groups				
	Control (Distilled Water)	BD.Me 10% Cream	BD.Me 20% Cream	Distachionate	Standard
TSP (mg g^−1^ FW)	3.29 ± 0.01	3.26 ± 0.01	2.62 ± 0.02	2.15 ± 0.14	1.92 ± 0.12
Catalase enzyme (U/mg protein)	23.30 ± 0.20	23.79 ± 0.20	28.81 ± 0.16	35.45 ± 0.25	44.32 ± 0.08
Suboxide dismutase enzyme (U/mg protein)	15.11 ± 0.01	15.24 ± 0.02	16.70 ± 0.06	28.10 ± 0.05	19.49 ± 0.08

Values shown are mean ± SD of BD.Me in antioxidant enzymes measurement (*n* = 6).

**Table 6:** Percent wound contraction of wounded skin area of excision wound healing model after receiving treatment on various days.

Excision Wound Healing Model						
Groups	Days					
	0	3rd	7th	10th	14th	21st
Control	0.0 ± 0.0	1.72 ± 0.61	19.0 ± 0.26	66.8 ± 1.21	81.4 ± 0.45	-
BD.Me 10% Cream	0.0 ± 0.0 ns	3.85 ± 0.85 ns	17 ± 0.36 ns	72.2 ± 1.17 *	88.7 ± 0.35 ***	-
BD.Me 20% Cream	0.0 ± 0.0 ns	6.46 ± 0.98 **/ns	29 ± 2.52 */**	79.2 ± 0.88 ***/**	92.4 ± 0.58 ***/**	-
Novel Distachionate	0.0 ± 0.0 ns	8.2 ± 0.04 ***/ns	32.6 ± 0.40 **/ns	78.5 ± 0.75 ***/ns	93.2 ± 0.55 ***/**	-
Standard Contractobex^®^	0.0 ± 0.0 ns	9.5 ± 1.02 ***/ns	30.8 ± 3.55 **/ns	81.3 ± 1.93 ***/ns	96.1 ± 0.92 ***/**	-

Values shown are mean ± SEM of BD.Me in excision wound healing study (*n* = 6) model. The values are * = *p* < 0.05 (significant), ** = *p* < 0.01 (more significant), *** = *p* < 0.001 (highly significant), and ns = *p* > 0.05 (non-significant), respectively.

**Table 7:** Percent wound contraction of wounded skin area of burn wound healing model after receiving treatment on various days.

Burn Wound Healing Model						
Groups	Days					
	0	3rd	7th	10th	14th	21st
Control (Base cream)	0.0 ± 0.0	−19.4 ± 0.74	24.3 ± 1.30	33.1 ± 2.97	46.2 ± 3.29	72.4 ± 0.85
BD.Me 10% Cream	0.0 ± 0.0 ns	−19.9 ± 1.16 ns	32.9 ± 2.66 *	42.3 ± 4.11 ns	58.8 ± 2.50 *	82.6 ± 1.12 ***
BD.Me 20% Cream	0.0 ± 0.0 ns	−17.5 ± 0.67 ns/ns	36.5 ± 1.45 ***/ns	45.9 ± 2.11 */ns	71.0 ± 2.11 ***/*	87.2 ± 0.99 ***/**
Novel Distachionate	0.0 ± 0.0 ns	−17.2 ± 0.81 ns/ns	28.5 ± 1.65 ***/ns	49.1 ± 3.10 ***/ns	67.2 ± 1.45	90.1 ± 0.55 ***/ns
Standard Contractobex^®^	0.0 ± 0.0 ns	−16 ± 1.21 ns/ns	38.2 ± 1.39 ***/ns	56.7 ± 2.46 ***/ns	70.5 ± 2.66	88.6 ± 0.84 ***/ns

Values shown are mean ± SEM of BD.Me in burn wound healing study (*n* = 6) model. The values are considered as * = *p* < 0.05 (significant), ** = *p* < 0.01 (more significant), *** = *p* < 0.001 (highly significant), and ns = *p* > 0.05 (non-significant), respectively.

**Table 8:** Anti-inflammatory effect of *Breynia distachia* methanol extract and distachinoate on carrageenan-induced edema model (mean ± SEM).

Group	Dose mg Kg^−1^	Increase in Paw Volume (mL)				
		1 h	2 h	3 h	4 h	5 h
Saline	-	0.680 ± 0.040	0.690 ± 0.040	0.700 ± 0.030	0.710 ± 0.030	0.720 ± 0.040
BD.ME	50	0.590 ± 0.030 (16.14%)	0.540 ± 0.040 (21.15%)	0.490 ± 0.030 (30.11%)	0.500 ± 0.040 (31.40%)	0.520 ± 0.040 (31.35%)
Distachionate	5	0.470 ± 0.054 (32.82%)	0.490 ± 0.051 (33.34%)	0.370 ± 0.040 ** (49.86%)	0.320 ± 0.060 ** (54.12%)	0.310 ± 0.050 ** (58.25%)
Indomethacin	5	0.230 ± 0.030 ** (68.92%)	0.200 ± 0.021 ** (72.61%)	0.180 ± 0.023 ** (75.34%)	0.190 ± 0.027 ** (72.47%)	0.190 ± 0.033 ** (74.33%)

% Protection is given in parenthesis. **: *p* < 0.01 compared with negative control group receiving saline.

**Table 9:** Histopathological findings of skin biopsy in punch model wound healing study.

Treatment	Scab Formation	Complete Reepithelization	Subepidermal Tissue Fibroblast	Granulated Tissues	Blood Vessel	Inflammatory Cell Type	Inflammation Intensity	Epidermal Changes
Control	−	−	−	+	−	lymphocytes	2	−
BD.Me 10% cream	−	−	−	−	+	Lymphocytes	1	−
BD.Me 20% cream	−	−	−	−	−	Neutrophils	1	−
Standard	−	−	−	−	−	lymphocytes	0	−

Where (+) indicates presence and (−) indicates absence and scoring for histopathological findings is indicated as (0) = none, (1) = mild, and (2) = moderate.

**Table 10:** Histopathological findings of skin biopsy in burn model wound healing study.

Treatment and Groups	Scab Formation		Complete Reepithelization		Sub-Epidermal Tissue Fibroblast		Granulated Tissues		Blood Vessel		Inflammatory Cell Type		Intensity of Inflammation		Epidermal Changes	
	14	21	14	21	14	21	14	21	14	21	14	21	14	21	14	21
Control	+	+	−	+	−	−	+	+	−	−	−	Lymphocyte	2	1	+	−
BD.Me 10% cream	+	−	+	+	−	−	+	−	−	−	−	−	1	−	+	−
BD.Me 20% cream	+	−	+	+	−	−	+	−	−	−	Neutrophil	−	1	−	−	−
Standard	−	−	+	+	−	−	−	−	−	−	−	−	−	−	−	−

Where (+) indicates presence and (−) indicates absence and scoring for histopathological findings is indicated as (0) = none, (1) = mild, and (2) = moderate.

## Data Availability

Requests to access the datasets should be directed to the corresponding author.

## Supplementary Materials

The following supporting information can be downloaded at: https://www.mdpi.com/article/10.3390/molecules27082596/s1, Experimental Section and Table S1: Summary of different solvent front scheme used in TLC analysis of *BD.Me.*

## Sample Availability

Samples of the compound are available from the first author at GCUF.
