# Supplementary material for: A Novel Distachionate from Breynia distachia Treats Inflammations by Modulating COX-2 and Inflammatory Cytokines in Rat Liver Tissue
Source: Molecules. 2022 Apr 18;27(8):2596. doi: 10.3390/molecules27082596 (PMC9027007; doi:10.3390/molecules27082596)
Supplement: Supplementary file 1 [file molecules-27-02596-s001.zip › molecules-1600227-supplementary.pdf]

Supplementary Materials

A Novel Distachionate from *Breynia distachia* Treats Inflammations by Modulating COX-2 and Inflammatory Cytokines in Rat Liver Tissue

Malik Saadullah [1], Muhammad Asif [2], Arshad Farid [3,*], Faiza Naseem [4], Sheikh Abdur Rashid [4,*], Shakira Ghazanfar [5], Muhammad Muzammal [3], Sohail Ahmad [3], Yousef A. Bin Jardan [6], Huda Alshaya [7], Muhammad Hamzah Saleem [8], Shafaqat Ali [9], Charles Oluwaseun Adetunji [8] and Sania Arif [1]

[1] Department of Pharmaceutical Chemistry, Government College University Faisalabad *38000*, Pakistan; maliksaadullah@gcuf.edu.pk (M.S.); arifsania456@gmail.com (S.A.)
[2] Faculty of Pharmacy, Islamia University of Bahawalpur, Bahawalpur 63100, Pakistan; asif_pharmacist45@yahoo.com
[3] Gomal Center of Biochemistry and Biotechnology, Gomal University, Dera Ismail Khan 29050, Pakistan; mustafamuzammal1@yahoo.com (M.M.); sohailbiotech383@gmail.com (S.A.)
[4] Department of Pharmaceutics, Faculty of Pharmacy, Gomal University, Dera Ismail Khan 29050, Pakistan; faizanaseem17@gmail.com
[5] National Institute for Genomics Advanced Biotechnology, National Agricultural Research Centre, Park Road, Islamabad 45500, Pakistan; shakira_akmal@yahoo.com
[6] Department of Pharmaceutics, College of Pharmacy, King Saud University, Riyadh 11451, Saudi Arabia; ybinjardan@ksu.edu.sa
[7] Department of Cell and Molecular Biology Program, University of Arkansas, Fayetteville, NC 72701, USA; hmalshay@uark.edu
[8] College of Plant Science and Technology, Huazhong Agricultural University, Wuhan 43070, China; saleemhamza312@webmail.hzau.edu.cn (M.H.S.); adetunji.charles@edouniversity.edu.ng (C.O.A.)
[9] Department of Biological Sciences and Technology, China Medical University, Taichung 40402, Taiwan; shafaqataligill@yahoo.com
[10] Applied Microbiology, Biotechnology and Nanotechnology Laboratory, Department of Microbiology, Edo State University Uzairue, Edo State 300271, Nigeria
* Correspondence: arshadfarid@gu.edu.pk (A.F.); sheikhabdurrashid11@gmail.com (S.A.R.)

Phytochemical Analysis Test for Flavonoids Alkaline Reagent Test

Small quantity of extract was added in a clean test tube, then 2 ml of 2 % NaOH (sodium hydroxide) solution was added in test tube, yellow color appeared. After that, a small quantity of HCl (hydrochloric acid) was added in it, the yellow color disappeared, this confirmed the presence of flavonoids.

Sulphuric Acid Test

In a test tube, containing small quantity of crude extract, 2 mL of concentrated H_2SO_4 (sulphuric acid) was added along the wall of test tube. The appearance of orange color ring indicated the presence of flavonoids.

3Lead Acetate Test

In a test tube, containing a small quantity of crude extract, 2-4 mL of lead acetate was added. The appearance of white precipitate confirmed the presence of flavonoids.

Test for Carbohydrates Iodine (I_2) Test

Freshly prepared iodine solution (2-3 drops) was added in a clean test tube which contained crude extract. The presence of carbohydrates was confirmed by the formation of purple black color.

Molisch Reagent Test

In a neat and clean test tube crude extract was added, then 2 mL of concentrated sulphuric acid (H_2SO_4) was added (along the wall of test tube to avoid contact with the

acid). The appearance of a violet ring at the interphase of the reaction mixture confirmed the presence of carbohydrates.

Benedict's Reagent Test

In a neat and clean test tube crude extract was added, then 2 mL of Benedict's reagent was added in test tube. Then test tube is heated until boiling of the mixture. The appearance of reddish brown color confirmed the presence of carbohydrates.

Fehling's Reagent Test

In a clean test tube crude extract was added, then both Fehling's A and B solution of about 2 mL was added. Then, the solution was boiled, the formation of brick color at bottom confirmed the presence of reduced sugars.

Test for Alkaloids

Small amount of extract was added in a test tube and 1% HCl (hydrochloric acid) solution was added. The test tube was heated, then mixture was divided in two separate test tubes in equal volume, then Mayer's and Wagner's reagent was added in separate test tubes. The presence of turbid solutions in both test tubes confirmed the presence of alkaloids.

Test for Tannins

Small quantity of extract was added in a neat test tube, then 2 mL of 2 % solution of ferric chloride ($FeCl_3$) was added. The presence of tannins was confirmed by observing a color change from black or blue to green.

Bromine (Br_2) Water Test

Small amount of crude extract was taken and 2 mL of bromine water was added. The presence of tannins was confirmed by discoloration of bromine.

Test for Proteins Coagulation Test

2-3 mL of distilled water was added in a small amount of plant extract in a test tube. Then the aqueous mixture was heated. The presence of coagulation at the end of test tube confirmed the existence of proteins in the plant extract.

Ninhydrin Solution Test

In a beaker, 2% Ninhydrin solution was prepared. Small amount of crude extract and 2ml of Ninhydrin solution was added in a neat test tube, the appearance of violet color was a sign of the presence of proteins in the extract sample (Ramamurthy and Sathiyadevi 2017).

Test for Terpenoids

2 mL of chloroform was added in the crude plant extract and the evaporation of chloroform was waited. Then 2 mL of conc. sulphuric acid was added. Mixture was heated for 2 minutes, the presence of terpenoids in the plant extract was confirmed by the appearance of greyish or intense color.

Test for Saponins

A small amount of extract was added in a clean test tube. Then 5 mL of distilled water was poured in it and the tube was continuously shaked. The presence of saponins was confirmed by the formation of a stable foam at top of the mixture.

Test for Glycosides

Salkowski's Test

A clean test tube was selected and small amount of crude extract was taken. Acetic acid (2 mL) and chloroform (2 mL) was mixed in test tube. Then, the test tube was cooled on cold water. Then concentrated H_2SO_4 (sulphuric acid) was added alongside of the test tube. The presence of glycosides was confirmed by the observation of color change from violet to blue to green color.

Keller Kiliani Test

Crude extract was taken in a test tube then 2-3 drops of 2 % ferric chloride ($FeCl_3$) solution and 2 mL of acetic acid solution was added in a test tube. After mixing, the mixture was transferred in another test tube which contained concentrated H_2SO_4 (sulphuric acid). The presence of a brown ring at interphase confirmed the presence of glycosides. (Gul, Jan et al. 2017).

Trace Metal Analysis

To screen out trace metals in plant extract acid digestion method was used. In a round bottom flask 1 g of *BD.Me* extract was added. The mixture of hydrochloric acid and nitric acid in ratio of 1:3 was added in a flask for the preparation of aqua-regia (HCl: HNO_3) reagent. Then extract and 10-15 mL of distilled water (to avoid evaporation of nitric acid fumes) and 15 mL of aqua-regia reagent was added in round bottom flask. Then flask was attached with the condenser and placed on hot plate for 2 h at 50-60 ^{o}C. It was concentrated to obtain 2-3 mL volume. Then cooled and filtered by using Whatman filter paper in a clean beaker. It was diluted with distilled water upto 100 mL and then placed this sample solution in a separate clean bottle for analysis. Trace metal analysis was performed by using atomic absorption spectrophotometer. By using commercially available stock, standards of metals copper (Cu), zinc (Zn), iron (Fe), magnesium (Mg), calcium (Ca), manganese (Mn) were prepared and diluted (upto 1000 ppm) with purified deionized water or distilled water. Glass apparatus was soaked with 8 N nitric acid overnight and washed with distilled water several times before use.

Total Phenolic Content (TPc) Assay

Folin Ciocalteu or Folin's Denis Reagent Test or Gallic Acid Equivalence Method

This study was conducted to find out the phenolic antioxidants or polyphenol contents of *Breynia distachia*. Dilution of Fc was performed by taking 5 mL Fc reagent in 45 mL distilled water, then 100 µL of Fc was used in the assay. The stock solution (10 mg/mL) of sample extract, solution of 7.5 % sodium carbonate (Na_2CO_3) and standard (Gallic Acid) was prepared in this assay. Serial dilutions of gallic acid was done having concentration (1000 µg/mL-7.81 µg/mL). In a microtitration plate 20 µL of plant extract and each standard dilution along with 80 µL of sodium carbonate were loaded in each well, then 100 µL of Fc reagent was added in each well. This assay was performed in triplicates. Control having (80 µL Na_2CO_3 + 20 µL distilled water + 100 µL Fc) and blank (200 µL distilled water) was also used. These microplates were incubated for 2 h in the dark, then read through Elisa microplate reader at 750 nm. Standard curve of gallic acid was obtained and results were expressed in µmol/mL (Samad, Asif et al. 2018).

Thin Layer Chromatography (TLC)

TLC process was performed in three steps (spotting, plate development and analysis or visualization). In 1st step, thin defined strip of silica gel plate was cut with a sharp sassier and a dot was marked at 1 cm (above from end) and 9-10 cm lengthwise on this plate, then placed a drop (less than 1 mm) of plant extract (stock 10 mg/mL) at the center of drawn line with the help of capillary tube and allowed to dry. In 2nd phase, different ratios of mobile phase were poured in the chromatographic tank and allow to saturate for 5 minutes, then the plate was placed in the tank straight in vertical position (making sure the spot did not immerse in the mobile phase). The lid was placed on the tank, when the mobile phase reached upto the upper line then strip was withdrawn and left to dry. Then,

via spray gun, the Godin's reagent, and 10 % sulphuric acid (H_2SO_4) were sprayed on the plate. In 3rd phase strip was visualized under ultraviolet lamp (Model UVGL-25, Multi-band UV-254 nm, 230 V, 50/60 Hz, 012 AMPS @ 220 V, 50 Hz, USA) and spots of different colors were circled then plate was heated at 100 ^{o}C in oven for 3 min. The appeared spots of different patterns were indicated with led pencil (Kumar, Jyotirmayee et al. 2013).

Table S1. Summary of different solvent front scheme used in TLC analysis of *BD.Me.*

Solvent Systems	Ratio of Solvents
Chloroform : Methanol : Water	100 : 35 : 3
Chloroform : Methanol : Water	80 : 20 : 2
Chloroform : Methanol : Water	65 : 35 : 5
Chloroform : Methanol : Water	8.5 : 3.5 : 0.5
Ethyl acetate : Methanol : Water	100 : 14 : 7
Ethyl acetate : Methanol : Water	7 : 1 : 0.5
Ethyl acetate : Methanol	10 : 2.5
n-hexane : Ethyl acetate	9 : 3
n-hexane : Ethyl acetate	8 : 3
n-hexane : Ethyl acetate	7 : 3

(R_f) value was calculated by following formula:

$$Rf = Distance\ travelled\ by\ compounds / Distance\ travelled\ by\ mobile\ phase$$

Antioxidant Assays

Radical scavengering potential of the *BD.Me* was done by using the ABTS (2, 2-azino-bis3ethylbenzothiazoline-6- sulphonic acid) DPPH (2, 2-diphenyl-1- picrylhydrazyl), and FRAP (Ferric reducing antioxidant powder) antiradical assays.

DPPH Antiradical Assay

For this, stock solution of plant extract and standard (10 mg/mL) as well as DPPH (200mM/L, 4mg/50 mL) was prepared. Six serial dilution (200 μg/mL-3.125 μg/mL) from stock solution of plant extract and standard (ascorbic acid) having concentration 25 μg/mL to 0.781 μg/mL were prepared. Then, in 96-well microtitration plate, 100 μL of each dilution of extract and standard was filled in every well along with control (100 μL ethanol + 100 μL DPPH) and blank of (200 μL) was added. Then, in darkness, 100 μL of DPPH reagent was added in each well. This assay was performed in triplicates. Plates was incubated for 30 minutes at room temperature. Free radical scavenging activity was confirmed by observing the change of color from violet to yellow. By Elisa microplate reader (at 534 nm), the absorbance of sample containing microplate was checked.

Percent radical scavenging activity is calculated by:

$$\%RSA = (1 - (Abs.\,sample - Abs.\,of\ blank) / Abs.\,of\ control - Abs.\,of\ blank)) \times 100$$

Regression line and IC_{50} were calculated by plotting graph between concentration (μg/mL) and absorbance of sample and standard separately (Mohamed, Asif et al. 2019).

ABTS antioxidant Assay

The solution of ABTS (14 mmol/L), and potassium persulphate (4.9 mmol/L) was prepared, mixed in equal volume and stored at room temperature upto 16-24 h. Then, 1 mL of above mixture and 40 mL of deionized water (to make the final dilute ABTS working solution) was added in separate container. Same procedures as described for DPPH assay

were conducted but the plate was incubated for 6 min and absorbance was measured at 734 nm. IC50 was calculated as explained in DPPH assay (Mohamed, Asif et al. 2019).

FRAP Antioxidant Assay

Solution of 0.2 M acetic acid and sodium acetate was made. 46.3 mL of acetic acid and 3.7 mL of sodium acetate was added to form the acetate buffer of 300 Mm/L concentration having pH 3.6. pH was adjusted by adding sodium hydroxide (NaOH) or hydrochloric acid (40 mM/L, 200 mL). Then 10 mmol/L solution of TPTZ and 20 mmol/L of $FeCl_3$ were prepared. Finally, 10 mL of acetate buffer was added in 1 mL of TPTZ and 1 mL of $FeCl_3$ (10:1:1), FRAP solution was formed. Six serial dilutions of standard ferrous sulphate 250 μg/mL to 7.812 μg/mL were prepared ,whereas extract was used directly from stock (10 mg/mL). Incubated for 8 min, and measured the absorbance at 600 nm. Result of FRAP assay was showed as nmol Fe^{+2} eq. per gram fresh weight (Mohamed, Asif et al. 2019).

Antioxidant Enzymes

The Catalase activity was measured with hydrogen peroxide, used as substrate. The rate of inhibition in photo-reduction of nitroblue tetrazolium (NTB) was calculated by evaluating superoxide dismutase activity. This method must contain at least 100 μL of sample (diluted between 5-100 μg of protiens) in one assay tube. In this activity the reaction mixture used must have 100 μL crude extract, 50 mM sodium phosphate buffer (pH=7.6), 0.1 mM EDTA, 50 μM NTB, 50 mM sodium carbonate, 10 μL of riboflavin and 12 mM L-methionine. Then, the mixture is placed in front of white light at room temperature for 15 min. Protein concentration in serum of rats was analyzed by Subramanian method. Bovine serum albumin was used as standard. In this method, at least 100 μL of sample (diluted between 5-100 μg of protiens) must be contained in one assay tube. As standard Bovine serum albumin was used. By adding 2 mg/mL of bovine serum albumin in 1000 μL volume standard was prepared which must be in the range of 200-2000 μg. Then, the mixture containing 4 mL dye reagent was incubated for 10 min. By using the spectrophotometer, absorbance was calculated at 240 nm, 595 nm and 520 nm, respectively (Wheeler, Salzman et al. 1990).

Study Design

Group-I (Standard): The Albino rats of this group acted as positive control and received contractobex® gel or any heparin containing cream.

Group-II (10 % treatment): This group received 10% extract cream. It was made by using topical gel base as vehicle.

Group-III (20 % treatment): The rats of this group were pretreated with 20% extract cream by using base cream as vehicle.

Group-IV (Control): Rats of this group acted as negative control by using distilled water 10 mL/kg.

For anti-inflammatory study, 100 mg/kg and 200 mg/kg of *BD.Me* was administered to group II and III, while indomethacin (standard) 10 mg/kg was given to group IV orally.
